# *Dnmt3a* deletion cooperates with the *Flt3/ITD* mutation to drive leukemogenesis in a murine model

**DOI:** 10.18632/oncotarget.11986

**Published:** 2016-09-12

**Authors:** Jennifer L. Poitras, Diane Heiser, Li Li, Bao Nguyen, Kozo Nagai, Amy S. Duffield, Christopher Gamper, Donald Small

**Affiliations:** ^1^ Department of Oncology, Johns Hopkins University School of Medicine, Baltimore, MD, USA; ^2^ Department of Pathology, Johns Hopkins University School of Medicine, Baltimore, MD, USA; ^3^ Department of Pediatrics, Johns Hopkins University School of Medicine, Baltimore, MD, USA

**Keywords:** FLT3, internal tandem duplication, DNMT3a, acute myeloid leukemia, mouse model

## Abstract

Internal tandem duplications of the juxtamembrane domain of FLT3 (FLT3/ITD) are among the most common mutations in Acute Myeloid Leukemia (AML). Resulting in constitutive activation of the kinase, FLT3/ITD portends a particularly poor prognosis, with reduced overall survival and increased rates of relapse. We previously generated a knock-in mouse, harboring an internal tandem duplication at the endogenous *Flt3* locus, which develops a fatal myeloproliferative neoplasm (MPN), but fails to develop acute leukemia, suggesting additional mutations are necessary for transformation. To investigate the potential cooperativity of FLT3/ITD and mutant DNMT3A, we bred a conditional *Dnmt3a* knockout to a substrain of our *Flt3/ITD* knock-in mice, and found deletion of *Dnmt3a* significantly reduced median survival of *Flt3^ITD/+^* mice in a dose dependent manner. As expected, pIpC treated *Flt3^ITD/+^* mice solely developed MPN, while *Flt3^ITD/+^*;*Dnmt3a^f/f^* and *Flt3^ITD/+^*;*Dnmt3a^f/+^* developed a spectrum of neoplasms, including MPN, T-ALL, and AML. Functionally, FLT3/ITD and DNMT3A deletion cooperate to expand LT-HSCs, which exhibit enhanced self-renewal in serial re-plating assays. These results illustrate that DNMT3A loss cooperates with FLT3/ITD to generate hematopoietic neoplasms, including AML. In combination with FLT3/ITD, homozygous Dnmt3a knock-out results in reduced time to disease onset, LT-HSC expansion, and a higher incidence of T-ALL compared with loss of just one allele. The co-occurrence of FLT3 and DNMT3A mutations in AML, as well as subsets of T-ALL, suggests the *Flt3^ITD/+^*;*Dnmt3a^f/f^* model may serve as a valuable resource for delineating effective therapeutic strategies in two clinically relevant contexts.

## INTRODUCTION

Large scale genomic studies have revealed the complex and heterogeneous genomic underpinnings of Acute Myeloid Leukemia (AML), identifying *FLT3* as the most frequently mutated gene in the disease, occurring in ~30% of AML patients.[1–3] Internal tandem duplication (ITD) of the juxtamembrane domain is the most common of these mutations, and predicts poor clinical outcomes.[3] Previous studies in knock-in mice illustrate that *Flt3*I*^TD/+^* alone generates a myeloproliferative neoplasm (MPN) and is insufficient to drive leukemogenesis, suggesting additional mutations are necessary for full transformation.[4, 5] Global genomic sequencing studies have identified a substantial subset of patients in which *FLT3/ITD* and *DNMT3A* mutations are concomitantly present.[1, 3] Moreover, the co-occurrence of these mutations is significantly associated with adverse clinical outcomes.[3] Typically in AML patients, sequencing studies have demonstrated 8-13 mutations but it is unclear how many of these mutations are drivers versus passengers, leaving in doubt the number of mutations required for full transformation of primary cells.[6] Based on these observations, investigating the potential cooperativity of these mutations in a murine model serves to answer important questions regarding the underlying biology of the disease, while serving as a powerful drug discovery tool with the potential for significant clinical impact.

*DNMT3A* mutations are among the most common alterations in AML, just behind *FLT3/ITD*.[1, 2, 7] While AML-associated mutations have been identified throughout the body of the gene, the overwhelming majority are heterozygous missense mutations within the catalytic domain, often affecting Arginine 882.[8] *In vitro* studies suggest the R882H mutation leads to reduced methyltransferase activity, and acts in a dominant negative manner by impairing tetramer formation.[9–11]

In accordance with mounting evidence that *DNMT3A* mutations result in a loss of function, conditional knock-out mice have been used extensively to evaluate the effects of Dnmt3a loss on stem cell function and leukemia development.[12, 13] These mice harbor floxed *Dnmt3a* alleles (*Dnmt3a^f/f^*) and Mx1-Cre, which is induced upon injection with polyinosinic-polycytidylic acid (pIpC).[14] Recent work demonstrated that upon serial transplantation, *Dnmt3a* ablation in hemaotpoietic stem cells (HSC) promotes self-renewal and expansion of the LT-HSC pool, with progressive differentiation and methylation defects of downstream progeny.[13, 15] As DNMT3A mutations are thought to represent one of the earliest founding events in AML, these functional studies may explain how mutant DNMT3A initiates the disease, through expanding the primitive cell pool, thereby increasing the probability of acquiring additional deleterious mutations.[16, 17] Conversely, *Flt3/ITD* disrupts LT-HSC quiescence, resulting in depletion of this compartment.[18] Given the co-occurrence of *FLT3/ITD* and *DNMT3A* mutations in AML patients, we hypothesized that *Dnmt3a* deletion may cooperate with *Flt3/ITD* to induce leukemia, potentially through rescuing the LT-HSC depletion seen in *Flt3^ITD/+^* mice.

**Figure 1:**
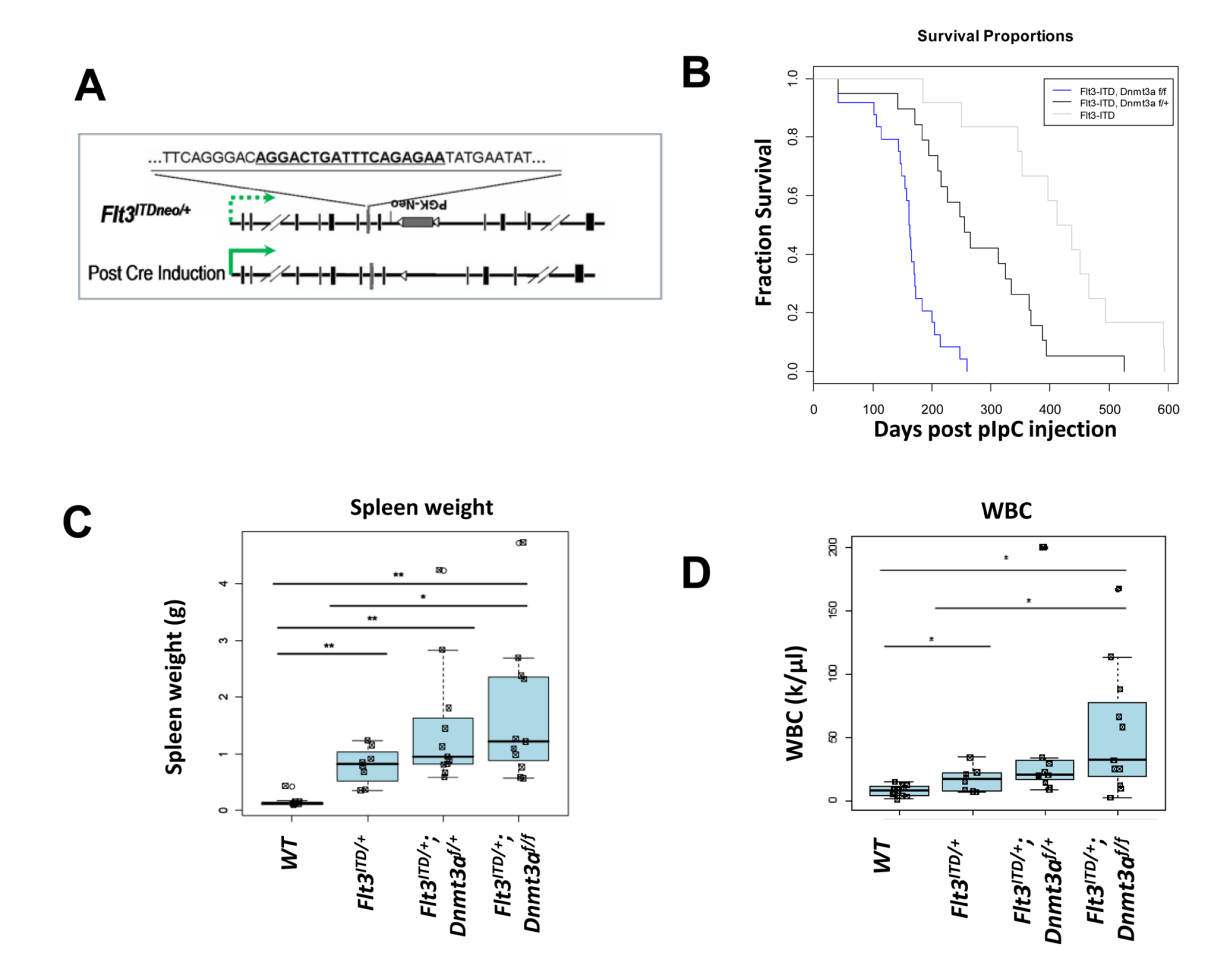
*Dnmt3a* deletion cooperates with *Flt3^ITD/+^* to shorten survival in a dose dependent manner **A.** Strategy for temporally controlling *Flt3/ITD* expression using a substrain of our *Flt3^ITD/+^* knock-in mice. The substrain (*Flt3^ITDneo/+^*) retains the PGK-Neo cassette inserted at the mutant allele (referred to hereafter as *Flt3^ITD/+^*). The presence of the cassette reduces expression of the mutant allele (dashed green arrow), and *Flt3^ITD/+^* expression is fully restored upon excision of the cassette following cre induction (solid green arrow). **B.** Kaplan-Meier Survival Curve. Median survival is 162 days for *Flt3^ITD/+^*; *Dnmt3a^f/f^* (*n* = 24), 260 days for *Flt3^ITD/+^*; *Dnmt3a^f/+^* (*n* = 20), and 412 days for *Flt3^ITD/+^* alone (*n* = 12). **C.** Spleen weights, and **D.** White blood cell counts (WBC) of mice developing myeloid neoplasms at the time of sacrifice. Wildtype (WT), FLT3/ITD alone (*Flt3^ITD/+^*), *Flt3^ITD/+^*; *Dnmt3a^f/+^*, *Flt3^ITD/+^*;*Dnmt3a^f/f^*. (*, *p* < 0.05; **, *p* < 0.01).

Since *DNMT3A* mutations seem to represent an initiating event, breeding *Dnmt3a^f/f^* mice to our *Flt3^ITD/+^* knock-in mice presents a significant challenge, as mutant *Flt3* is expressed at the earliest stages of hematopoeisis, preceding Dnmt3a deletion. To partially circumvent this issue, we used a substrain of our *Flt3^ITD/+^* knock-in mice which retains a floxed Neomycin (Neo) selection cassette from the initial targeting (*Flt3^ITDneo/+^*; referred to hereafter as *Flt3^ITD/+^* for simplicity's sake). The presence of the cassette greatly reduces expression of the mutant allele.[4] *Flt3^ITD/+^* expression is fully restored upon excision of the cassette following Cre induction, effectively “knocking in” the *Flt3/ITD* mutation and knocking out *Dnmt3a* simultaneously (Figure 1A). This novel approach allowed us to perform long term survival studies and disease characterization in a biologically relevant context.

## RESULTS

### Dnmt3a deletion reduces survival of Flt3^ITD/+^ knock-in mice in a dose dependent manner

In accordance with mounting evidence that *DNMT3A* mutations result in a loss of function, we used a mouse model harboring floxed *Dnmt3a* alleles (*Dnmt3a^f/f^*), and a Cre transgene under control of the type I interferon inducible Mx1 promoter (Mx1-Cre), which is activated upon injection with pIpC, inducing LoxP recombination and excision in hematopoietic cells.[9, 14, 19] These mice were bred to a substrain of our *Flt3^ITD/+^* knock-in mice, which retains a floxed PGK-Neo selection cassette from the initial targeting (Figure 1A). The presence of the cassette reduces transcription of the mutant allele, and full expression is restored upon Cre excision.[4] Since *DNMT3A* mutations are thought to represent one of the earliest events in leukemia development, this approach provides the unique advantage of temporally restraining *Flt3^ITD^* expression until *Dnmt3a* is lost; effectively “knocking in” the *Flt3^ITD^* mutation, and knocking out *Dnmt3a* simultaneously upon pIpC injection.[2, 16, 17] Mx1-Cre expressing mice harboring *Flt3^ITD/+^* and homozygous floxed *Dnmt3a* alleles (*Flt3^ITD/+^*;*Dnmt3a^f/f^*), heterozygous (*Flt3^ITD/+^*;*Dnmt3a^f/+^*), or wild type alleles (*Flt3^ITD/+^*), as well as littermate controls (*Flt3^+/+^*;*Dnmt3a^f/f^* or *Flt3^ITD/+^*;*Dnmt3a^f/+^* lacking the Mx1-Cre transgene) were injected with pIpC intraperitoneally at 8 weeks of age and monitored for disease development. Peripheral blood was collected every 1-2 months after injection to monitor changes in the white blood cell differential, and to collect genomic DNA to confirm effective loxP recombination (Figure S1).

Interestingly, deletion of *Dnmt3a* significantly reduced median survival of *Flt3^ITD/+^* mice in a dose-dependent manner, with median survival of 162 days and 256 days for *Flt3^ITD/+^*;*Dnmt3a^f/f^* and *Flt3^ITD/+^*;*Dnmt3a^f/+^*, respectively, irrespective of disease diagnosis (Figure 1B). Both genotypes confer a significantly shorter survival time compared to *Flt3^ITD/+^* mice alone, which have a median survival of 412 days, consistent with our previous findings.[4, 20, 21] *Dnmt3a* dosage was also associated with a number of other parameters, including splenomegaly (Figure 1C) and leukocytosis (Figure 1D). While the trending increase in spleen weight and WBC is evident, only *Flt3^ITD/+^* mice with complete loss of *Dnmt3a* (*Flt3^ITD/+^*;*Dnmt3a^f/f^*) displayed increases reaching statistical significance compared to *Flt3^ITD/+^* alone.

### Dnmt3a deletion cooperates with Flt3^ITD/+^ to induce a broad spectrum of neoplasms including AML and T-ALL

**Figure 2:**
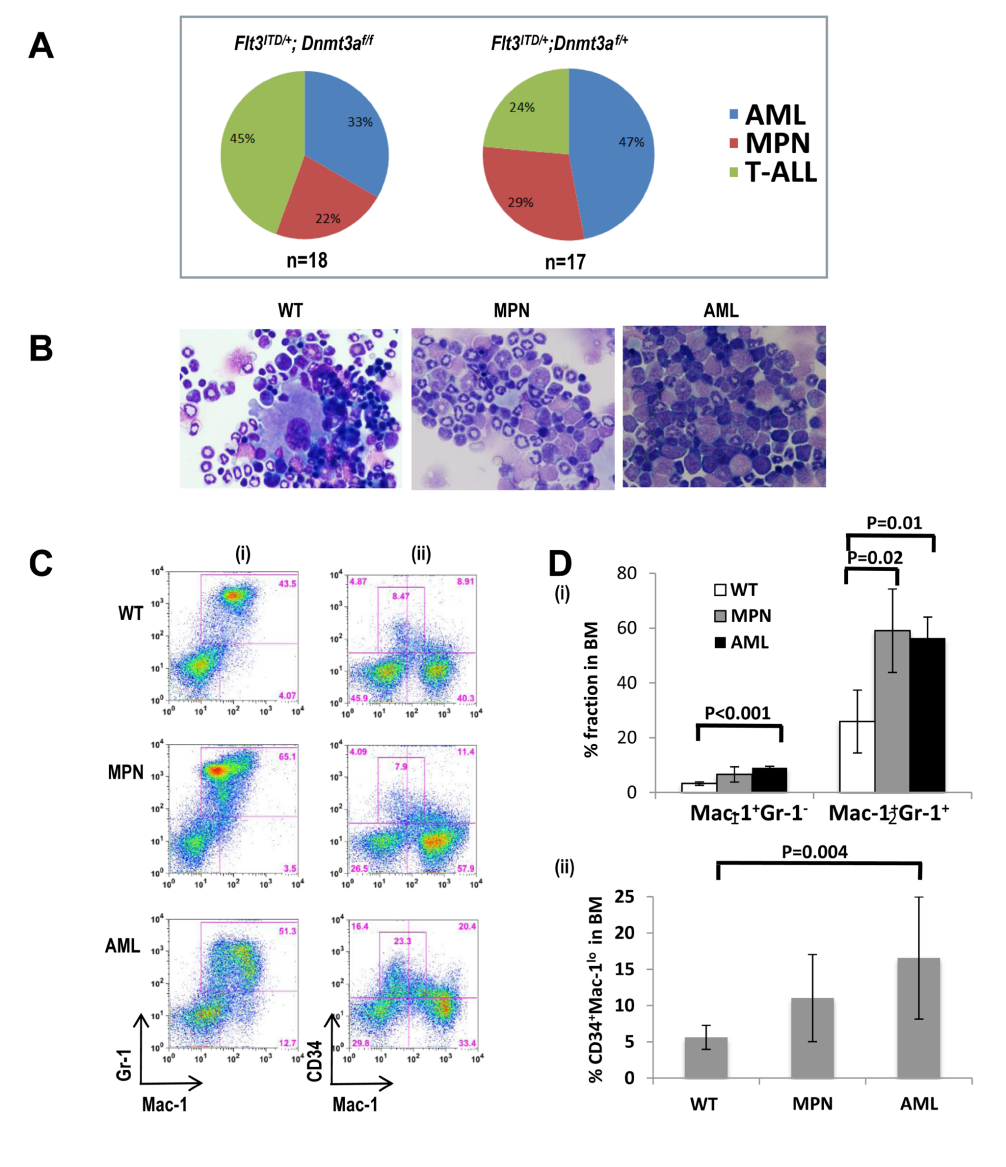
*Dnmt3a* deletion cooperates with *Flt3^ITD/+^* to induce a broad spectrum of neoplasms **A.** Disease distribution of *Flt3^ITD/+^*mice lacking both (left) or a single (right) *Dnmt3a* allele. **B.** Hematoxylin and Eosin stained bone marrow cytospins from a representative wild type mouse (WT, left column), a mouse with a Myeloproliferative Neoplasm (MPN, center column), and a mouse with Acute Myeloid Leukemia (AML, right column). **C.**, **D.** Flow cytometric analysis of bone marrow. (i) The MPN mouse displays the typical Mac1^+^Gr1^+^ expansion, while the leukemic mouse displays an abnormal Mac1^+^Gr1^lo/-^ population. (ii) The wild-type mouse displays the normal CD34 positivity, while AMLs exhibit increased immature CD34^+^cells which appears to be Mac1^lo^ CD34^+^. In this experiment, *n* = 5 mice for each group.

As expected from previous work, mice with *Flt3^ITD/+^* alone developed MPN, while loss of one or both *Dnmt3a* alleles cooperates with the *Flt3^ITD^* mutation to elicit the development of an MPN or an acute leukemia of varying lineages including AML and T lymphoblastic leukemia /lymphoma (T-ALL) (Figure 2A). Histological analysis of bone marrow derived from mice with myeloid diseases revealed a predominance of myeloid blasts and immature myeloid cells in AML samples (Figure 2B). While T-ALLs displayed variable immunophenotypes, including double or single positive for CD4 or CD8 (data not shown), the immunophenotype among myeloid mice was fairly uniform, displaying an increase in blasts that are CD34+ and Mac1^+/lo^, consistent with a myelomonocytic leukemia (Figure 2C, 2D).

**Figure 3:**
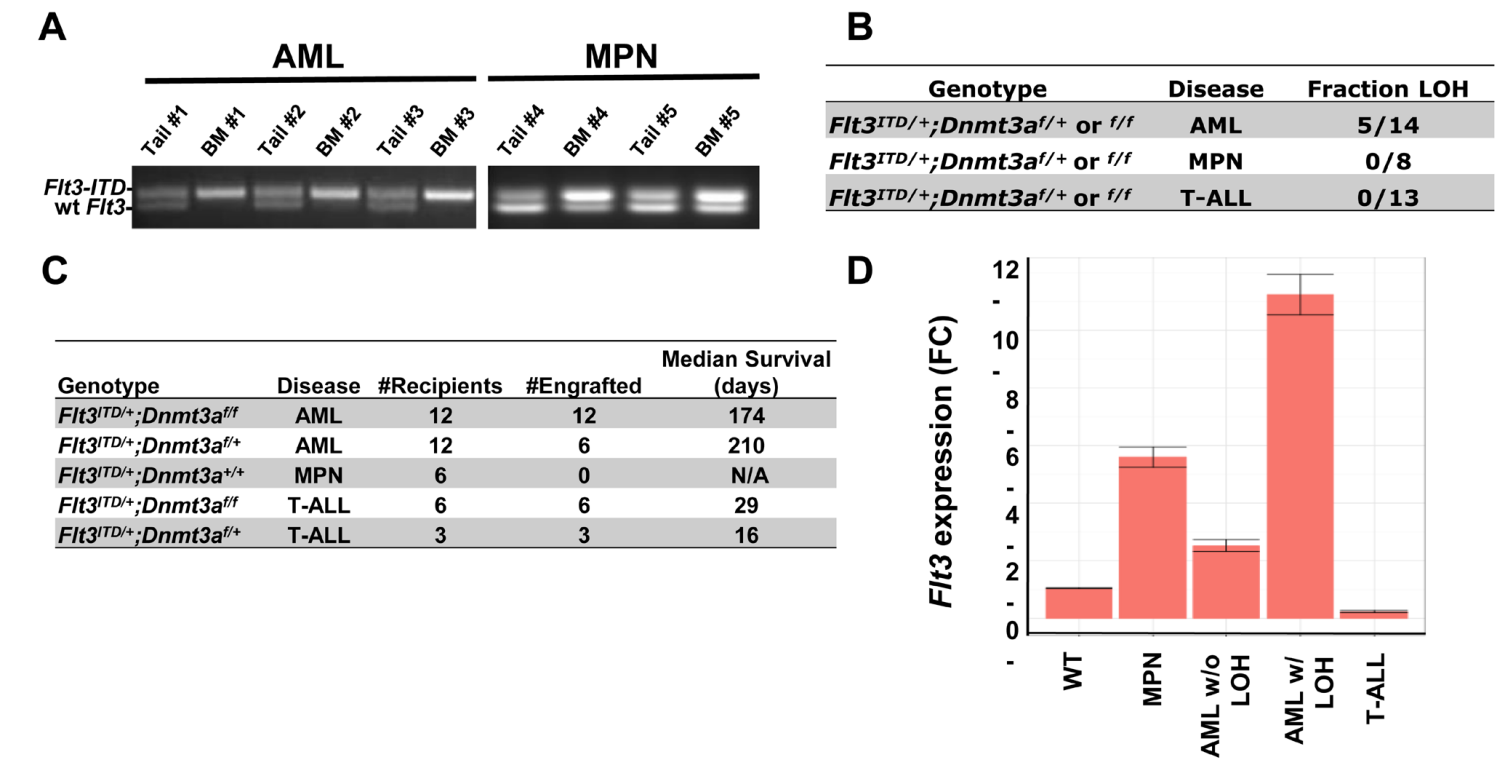
Loss of Heterozygosity (LOH) and *Flt3* expression are present in a disease specific manner **A.** Representative samples exhibiting bone marrow specific LOH at the *Flt3* locus. Primers flanking the ITD mutation were used to amplify genomic DNA prepared from bone marrow and matched tail tissue from representative AML mice (#1, #2 and #3) and MPN mice (#4 and #5). **B.** Summary of samples exhibiting LOH. Complete loss of the wild type *Flt3* allele occurs exclusively in mice developing AML, in both *Flt3^ITD/+^;Dnmt3a^f/f^*and *Flt3^ITD/+^;Dnmt3a^f/+^* genotypes. BM from some of the MPN mice demonstrated higher ITD *vs*. wt allelic ratio but no signs of complete loss of the wild-type allele. **C.** Survival summary of transplantation experiments. Diseases of donors and median survival of all engrafted recipients are noted. **D.** T-ALLs are driven by FLT3/ITD-independent factors. qPCR of leukemic tissue displays silencing of *Flt3* in lymphocytic leukemia samples. RNA was extracted from whole bone marrow of Wildtype (WT), *Flt3^ITD/+^;Dnmt3a^f/f^* mice developing MPN, AML without LOH, AML with LOH at the *Flt3* locus, or T-ALL. *N* = 5 mice per genotype in each experiment.

In FLT3/ITD+ AML, loss of heterozygosity of the wild type allele is sometimes observed, and is associated with poorer survival.[22–24] To investigate whether or not this phenomenon also occurred in our model, genomic DNA was extracted from bone marrow and matched tail tissue from leukemic mice, and PCR amplification was performed using primers flanking the ITD mutation (Figure 3A). Loss of the wild type allele was observed in 36% (5/14) of mice developing AML (both *Flt3^ITD/+^*;*Dnmt3a^f/f^* and *Flt3^ITD/+^*;*Dnmt3a^f/+^*), while no evidence of complete loss of wild-type allele was observed in any of the 13 mice developing lymphoid neoplasms and the 8 mice developing myeloproliferative neoplasms (Figure 3B). None of the 8 MPN BM samples showed complete loss of the wild-type allele. However, it is notable that 3 of the MPN BM samples exhibited a higher ITD *vs* wild-type allelic ratio. This might indicate that loss of wild-type allele is occurring in some but not all of the BM cells, perhaps on their way to developing AML (Figure 3B). These findings are consistent with previous studies in *Flt3^ITD/+^* knock-in mice, where loss of the wild type allele is restricted to AML, and absent in T-ALL, suggesting a selective advantage for these events specifically in the myeloid lineage. [20, 25]

### T-ALL and AML samples are transplantable, with transplanted T-ALL exhibiting particularly aggressive disease

To investigate transplantability of primary neoplasms developing in our mice, cells from each leukemic donor were transplanted into three sublethally irradiated recipients and monitored for survival (Figure 3C). Engraftment and survival were variable based on disease subtype, as all 4 of the AML samples from *Flt3^ITD/+^*;*Dnmt3a^f/f^* donors engrafted, with a median survival of 174 days, while only 2 of 4 *Flt3^ITD/+^*;*Dnmt3a^f/+^* AML samples engrafted, displaying slightly prolonged (but not statistically significant) median survival. Transplantation of MPNs derived from *Flt3^ITD/+^* mice failed to engraft, consistent with previous findings.[4] In stark contrast, T-ALL samples from both *Flt3^ITD/+^*;*Dnmt3a^f/f^* and *Flt3^ITD/+^*;*Dnmt3a^f/+^* genotypes were much more aggressive, killing recipients within one month.

### T-ALLs in mice with FLT3-ITD and DNMT3A deletion exhibit repressed Flt3 expression

Previous sequencing endeavors have identified *DNMT3A* and *FLT3* as frequently mutated genes in AML, both individually and concurrently, and at lower frequencies in T-ALL.[26–28] While lymphoid leukemias haven't previously been observed in our *Flt3^ITD/+^* knock-in model, previous work has demonstrated that ~15% of *Dnmt3a^f/f^* mice develop T-ALL, with a median survival of 246 days, compared with 162 days in our *Flt3^ITD/+^*;*Dnmt3a^f/f^* mice.[12] Taken together, these findings suggest that perhaps the *Flt3/ITD* mutation is dispensable for maintenance of lymphoid malignancies in *Flt3^ITD/+^*;*Dnmt3a^f/f^* mice, but may play an important role in expanding the progenitor pool giving rise to these lymphoid neoplasms, thereby accelerating disease development.

To examine *Flt3* expression, we performed qPCR on whole bone marrow from wild type and *Flt3^ITD/+^*;*Dnmt3a^f/f^* mice with varying diagnoses. *Flt3* expression was elevated in myeloid neoplastic samples, especially those exhibiting LOH, with expression levels at about 11 fold higher than wild type bone marrow samples (Figure 3D). Interestingly, T-ALL samples expressed virtually no *Flt3*, supporting the hypothesis that once full transformation occurs in these cells, *Flt3* is turned off, as in normal lymphoid development.[29]

### Myeloid progenitors are expanded in double mutant mice at an early time point, underlying observed relative monocytosis and disease variability

Recent work has demonstrated that *Dnmt3a* deletion in HSC, promotes self-renewal and expansion of the LT-HSC pool, resulting in leukemia with incomplete penetrance and prolonged time to disease development. Conversely, *Flt3^ITD/+^* disrupts LT-HSC quiescence, resulting in depletion of this compartment, and failure to develop acute leukemia in the absence of cooperating mutations.[12, 13, 18] We hypothesized that enhanced self-renewal and expansion in the LT-HSC compartment conferred by *Dnmt3a* deletion might “rescue” the LT-HSC depletion seen in *Flt3^ITD/+^* mice, thereby increasing this primitive pool and the opportunity for additional mutations necessary to drive either myeloid or T cell leukemia to develop. To investigate the frequencies of progenitor populations well prior to disease onset, mice were sacrificed at 8 weeks post pIpC injection and the stem cell compartment was examined by flow cytometry.

**Figure 4:**
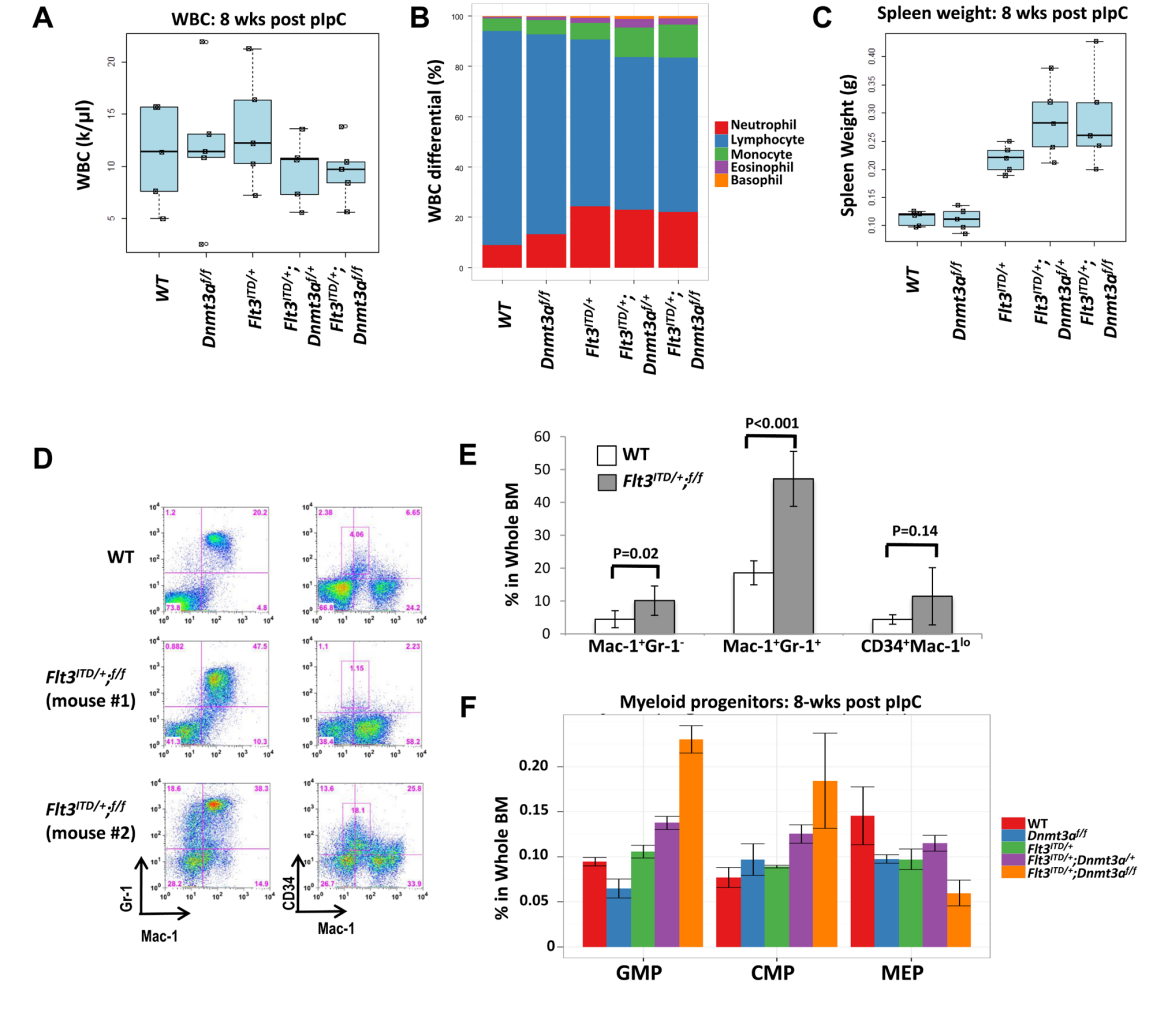
Early signs of disease development and myeloid expansion are present at 8 weeks post pIpC injection **A.** WBC remain within the normal range for all genotypes. **B.** Relative monocytosis is observed upon CBC, despite normal WBC. WT and *Dnmt3a^f/f^* exhibit normal leukocyte ratios, while *Flt3^ITD/+^* mice display an increase in percentage of neutrophils and concomitant decrease in percentage of lymphocytes. When these mice lose one or both *Dnmt3a* alleles, the phenotype is exaggerated with further decrease in relative lymphocytes and an increase in the percentage of monocytes. **C.** Spleen weight is increased only in mice harboring a FLT3/ITD mutation. Splenomegaly is further enhanced with loss of one or both Dnmt3a alleles. **D.**, **E.** Flow cytometric analysis of bone marrow at 8 weeks reveals abnormal patterns of myeloid maturation and CD34 expression. Two representative mutant mice are shown in D. *N* = 5 and 10 for the wild-type and *Flt3^ITD/+^*; *Dnmt3a^f/f^* groups, respectively. **F.** Shifts in the myeloid progenitor compartment are present. Wildtype (WT), *Dnmt3a* knockout alone (*Dnmt3a^f/f^*), FLT3/ITD alone (*Flt3^ITD/+^*), *Flt3^ITD/+^*; *Dnmt3a^f/+^*and *Flt3^ITD/+^*;*Dnmt3a^f/f^ (Flt3^ITD/+^;^f/f^)*. *N* = 5 mice per genotype in each experiment.

While overt leukemia was absent at this early time point, pathological changes were evident in the bone marrow and peripheral blood. None of the genotypes exhibited leukocytosis (Figure 4A), but relatively increased fractions of granulocytes and monocytes were seen, as expected, in *Flt3^ITD/+^* mice, and was exaggerated in the *Flt3^ITD/+^*;*Dnmt3a^f/+^* and *Flt3^ITD/+^*;*Dnmt3a^f/f^* mice, with a concomitant decrease in percent lymphocytes (Figure 4B). While *Dnmt3a* ablation alone had no effect on spleen size at 8 weeks, splenomegaly was noted in *Flt3^ITD/+^* mice, which was again more pronounced in double mutant mice (Figure 4C). Flow cytometric analysis of whole bone marrow displayed varying anomalies, including aberrant patterns of myeloid antigen and CD34 expression as well as expansion within the myeloid lineage (Figure 4D). This immunophenotypic variability at an early time point likely underlies the ultimate variability in neoplasms developing in moribund mice. Interestingly, while percentage shifts favoring myeloid cell types in the peripheral blood were similar between *Flt3^ITD/+^*;*Dnmt3a^f/+^* and *Flt3^ITD/+^*;*Dnmt3a^f/f^* mice, appreciable shifts in myeloid progenitor populations were only present in the *Flt3^ITD/+^*;*Dnmt3a^f/f^* bone marrow samples, where obvious increases in common myeloid (CMP) and granulocyte-macrophage (GMP) progenitors were observed with a decrease in megakaryocyte-erythrocyte progenitors (MEP) (Figure 4F).

### Loss of both copies of Dnmt3a is necessary to elicit LT-HSC expansion

**Figure 5:**
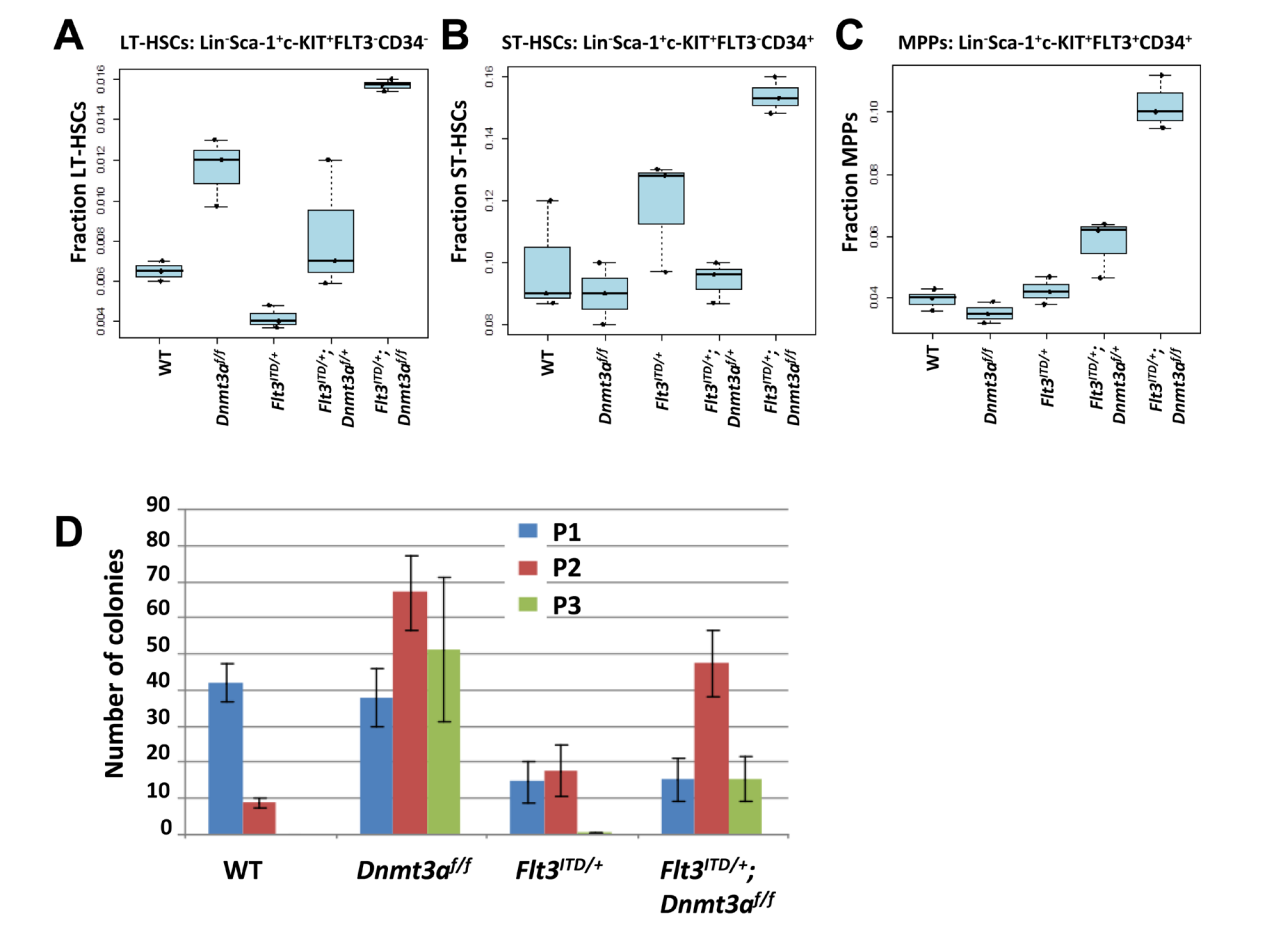
*Dnmt3a* deletion rescues the LT-HSC depletion phenotype seen in *Flt3^ITD/+^* knock-in mice **A.** LT-HSC, **B.** ST-HSC, and **C.** MPP populations were quantified by flow cytometric analysis of whole bone marrow of mice at 8 weeks post pIpC. *n* = 3 per genotype. **D.** Serial replating of Lineage negative bone marrow at 8 weeks post pIpC from 3 mice per genotype. Each plating was performed in triplicate. Wildtype (WT), *Dnmt3a* knockout alone (*Dnmt3a^f/f^*), FLT3/ITD alone (*Flt3^ITD/+^*), *Flt3^ITD/+^*; *Dnmt3a^f/+^* and *Flt3^ITD/+^*;*Dnmt3a^f/f^*.

Upon examining the stem cell compartment, we confirmed the previously described expansion in *Dnmt3a* knock-out mice and depletion in *Flt3^ITD/+^* mice. The data suggest that loss of one *Dnmt3a* allele is sufficient to restore the LT-HSC (Lin^−^ c-kit^+^ Sca-1^+^ Flt3^−^CD34^−^) pool of *Flt3^ITD/+^* mice to wild type proportions, while loss of both *Dnmt3a* alleles together with *Flt3/ITD* expression results in a dramatic expansion, beyond that of *Dnmt3a^f/f^* alone (Figure 5A). Given the previous findings that *Dnmt3a* deletion expands the LT-HSC compartment through enhanced quiescence, the observed minor decrease in the immediate downstream ST-HSC (Lin^−^ c-kit^+^ Sca-1^+^ Flt3^−^CD34^+^) (Figure 5B) and MPP (Lin^−^ c-kit^+^ Sca-1^+^ Flt3^+^CD34^+^) (Figure 5C) populations in *Dnmt3a^f/f^* mice is not surprising. Likewise, as aberrant exit from quiescence driven by *Flt3^ITD/+^* alone resulted in LT-HSC depletion, these cells are driven to mobilize and differentiate, as evidenced by an observed increase in the ST-HSC (Figure 5B) and MPP (Figure 5C) pools. Similar to the LT-HSCs, haploinsufficiency of *Dnmt3a* in *Flt3^ITD/+^*;*Dnmt3a^f/+^* mice was sufficient to bring the fraction of ST-HSCs back towards the wild-type levels. In contrast, loss of both *Dnmt3a* alleles in *Flt3^ITD/+^*;*Dnmt3a^f/f^* mice resulted in a dramatic increase in the ST-HSC compartment (Figure 5B). *Dnmt3a* deletion in the *Flt3^ITD/+^* context conferred an increase in the MPP population in a dose dependent manner (Figure 5C).

### Dnmt3a ablation enhances colony formation of Flt3^ITD/+^ bone marrow in serial replating experiments

After determining that *Dnmt3a* loss and the *Flt3/ITD* mutation do indeed cooperate to greatly expand the LT-HSC pool, we sought to investigate the mechanism underlying this observation. To assess self-renewal, lineage negative bone marrow cells were plated on methylcellulose, and serially re-plated twice more. The number of colonies was recorded before each re-plating. Since the methylcellulose used in these experiments contains cytokines directing myeloid differentiation, persistence of colony forming cells in the third plating suggests a block in differentiation and enhanced self-renewal. As expected, wild type bone marrow displays a significant decrease in colony formation at the secondary plating and an absence of colonies at the tertiary re-plating. In agreement with previous observations, *Dnmt3a^f/f^* cells continued to re-plate through the tertiary plating, exhibiting enhanced self-renewal. Only one tertiary re-plating colony was observed of all plates analyzed from *Flt3^ITD/+^* mice, congruent with disrupted quiescence (Figure 5D). However, when *Dnmt3a* loss was added to the *Flt3^ITD/+^* background, partial restoration of the enhanced self-renewal phenotype is observed (Figure 5D). These experiments suggest that the expansion in the LT-HSC pool of *Flt3^ITD/+^*;*Dnmt3a^f/f^* mice is achieved, at least in part, by enhanced self-renewal conferred by *Dnmt3a* deletion.

## DISCUSSION

Throughout the last decade, thorough analysis of the leukemia genome has generated an extensive compendium of AML-associated mutations.[1, 2] These data have formed the basis for subsequent functional studies in mice, using knock in, knock out, or transgenic strains. Mutation of the orthologous mouse gene represents the most biologically relevant experimental approach, yet many of the most common driver mutations do not, in isolation, lead to fully penetrant, rapidly fatal leukemias.[4, 5, 21, 30–32] These results are not surprising, as elegant studies dissecting the clonal evolution of leukemia indicate several mutations occur in concert, often chronologically, and are likely necessary for transformation.

Integrative genomic profiling has identified a significant proportion of AML patients with concomitant *FLT3-ITD* and *DNMT3A* mutations. Concurrently, these mutations stratify patients into a poorer prognostic category, predicting inferior clinical outcomes and overall survival.[3] It appears that neither FLT3/ITD nor DNMT3a mutations alone is sufficient for leukemic transformation. FLT3/ITD mutations have been shown to be a later event in leukemic clonal revolution.[33, 34] In contrast, loss of DNMT3a is one of the pre-leukemic events found in AML patients.[17, 34] In mouse, DNMT3A is highly expressed in HSCs enabling efficient differentiation through the epigenetic silencing of HSC regulatory genes.[13] Loss of DNMT3a promotes HSC self-renewal at the expense of differentiation. It is likely that the presence of the FLT3/ITD and DNMT3a mutations provides mouse HSCs with self-renewal and proliferative advantages. At the same time, hematopoietic differentiation is blocked as a result of aberrant methylation of HSC regulatory genes caused by DNMT3a loss.

While *Flt3/ITD* knock-in alone fails to fully transform and recapitulate human leukemia, ablation of *Dnmt3a* alone is sufficient to predispose HSCs to malignant transformation, resulting in a spectrum of neoplasms with a prolonged time to disease development.[12] We hypothesized that breeding *Flt3^ITD/+^* mice and the conditional *Dnmt3a* knock-out would result in shortened survival compared to either mutation alone, cooperating to drive AML development in a greater proportion of mice than *Dnmt3a^f/f^* alone.

It has recently been reported that the *Flt3*^ITD/ITD^*; Dnmt3a*^f/f^ mice spontaneously developed a fully penetrant AML while *Flt3*^ITD/+^;*Dnmt3a* knockout mice had a much longer disease latency and disease spectrum.[35] This suggests that the relative allelic ratio of ITD *vs*. wild type FLT3 is an important factor when evaluating the disease aggressiveness in the *FLT3/ITD DNMT3a* knockout double-mutant mice. Likewise, our data reported here confirm that indeed, *Flt3^ITD/+^* and bone marrow specific *Dnmt3a* deletion cooperate to result in shortened survival due to the development of fatal hematopoietic neoplasms, including AML and T-ALL. This is consistent with the recent finding of the development of AML and T-ALL in a similar *FLT3/ITD* and *DNMT3a*-KO double-mutant mouse model.[36] Interestingly, we also found that *Dnmt3a* dosage significantly impacts survival and the spectrum of neoplasms developing in *Flt3^ITD/+^* mice. The observation that loss of a single allele is sufficient to shorten survival and elicit leukemia development highlights the importance of *Dnmt3a* stoichiometry in maintaining appropriate hematopoietic stem cell function. This finding is especially relevant in light of a recent meta-analysis of the TCGA cohort, which revealed a significant focal loss of CpG methylation throughout the genomes of AML patients harboring *DNMT3A* mutations.[9] Mean beta values were also further reduced in DNMT3A^R882^ compared to DNMT3A^non-R882^ samples, supporting *in vitro* evidence that R882 mutations impair methyltransferase activity more severely than mutations at other residues. Future assessment and comparison of these *Flt3^ITD/+^;Dnmt3a^f/+^* and *Flt3^ITD/+^;Dnmt3a^f/f^* mice with human FLT3^ITD^;DNMT3A^non-R882^ and FLT3^ITD^;DNMT3A^R882^ AML methylomes may reveal differentially methylated loci that are dosage, rather than sequence context dependent.

Unlike its high frequency in AML, FLT3/ITD mutation is a rare event in human T-ALLs, with an incidence of 2% or less and no prognostic significance.[27, 37] On the other hand, DNMT3a mutations occur frequently in adult T-ALL, ranging from 16% to 26.3% and are associated with poor prognosis and short survival.[8, 27, 38] Also unlike AML, which is characterized by heterozygous *DNMT3A* mutations primarily in the methyltransferase domain, the gene is frequently biallelically inactivated in T-ALL, with mutations occurring throughout the body of the gene.[8] Homozygous ablation of *Dnmt3a* in our mice may mimic biallelic inactivation seen in patients, accounting for the incidence of T-ALL in these mice. While *Flt3* is virtually unexpressed in T-ALL derived from our double mutant mice, this does not preclude the possibility that FLT3/ITD is important for expansion of a lymphocytic progenitor, with cooperating somatic mutations and epigenetic changes favoring transformation within a later compartment, where FLT3 activity is dispensable.[29] The identification of FLT3 mutations in a subset of T-ALL patients raises important questions that warrant further investigation.[26, 27]

Variance in phenotype and delay in disease development may indicate that cells in the context of both mutations are still predisposed to transformation, but require additional mutations. Although leukemic mice meet the diagnostic criteria consistent with AML, including >20% blasts, inconsistent engraftment makes it difficult to perform transplantation experiments and *in vivo* drug treatments. A recent study in AML1-ETO driven murine leukemia illustrates the utility and power of cross referencing genomic data from murine studies with the enormous repository of publicly available data from human leukemia samples sharing the same genetic underpinnings.[33] Using a similar approach in our model, we hope to identify cooperative orthologous genomic events which we can introduce into *Flt3^ITD/+^;Dnmt3a^f/f^* bone marrow, thereby creating an increasingly relevant model of the disease, increasing aggressiveness and facilitating *in vivo* TKI and epigenetic combination therapy testing.

This model also presents a platform to answer important questions regarding DNMT3A dosage in the context of FLT3/ITD. Prior to leukemia formation, the incremental expansion of the LT-HSC pool indicates that differentially methylated loci important in maintaining quiescence can be determined. The data presented in this report demonstrate that conditional deletion of *Dnmt3a* and simultaneous “knock in” of *Flt3^ITD/+^*, cooperate to drive leukemia development at a faster rate than *Dnmt3a* loss alone. Loss of heterozygosity of the *Flt3* allele in our double mutant AMLs further substantiates our model as a powerful tool to study human leukemogenesis, as these events also spontaneously occur in FLT3/ITD+ patients. This model is a powerful tool that allows us to characterize the interplay between FLT3-ITD and DNMT3A mutations in the development and maintenance of both myeloid and lymphoid leukemias, and provides a novel clinically-relevant model for the development and evaluation of therapies for acute leukemia.

## MATERIALS AND METHODS

### Mice

Mice harboring floxed *Dnmt3a* alleles (*Dnmt3a^f/f^*) and a pIpC-inducible Mx1-Cre were bred to a substrain of our *Flt3^ITD/+^* knock-in mice which retain a floxed PGK-Neo cassette from the initial targeting (Figure 1A).[4, 14] *Dnmt3a* knock-out, and excision of the PGK-Neo cassette within the *Flt3^ITD/+^* allele, were achieved by two intraperitoneal injection of pIpC (250ug/mouse in PBS; Invivogen) every other day. Genomic DNA was prepared from peripheral blood eight weeks after pIpC injection to confirm loxP recombination at both alleles (Figure S1) Primers used for genotyping and confirmation of loxP recombination (Flox F & Flox R) can be found in Table S1. Diagnoses and classification of hematopoietic neoplasms were made based on the previously established Bethesda proposals.[39, 40] All animal experiments were performed according to protocols approved by the Animal Care and Use Committee of Johns Hopkins University in accordance with guidelines set forth by the National Institutes of Health.

### Complete peripheral blood cell count and cytology

Mice were monitored and sacrificed when they exhibited signs of disease development (lethargy, ruffled coat, abnormal complete blood count (CBC) differential). Peripheral blood was collected from the facial vein and subjected to complete blood cell counting, and a WBC differential was performed using the Hemavet950 system (Drew Scientific). Bone marrow cytospins and peripheral blood smears were stained using a modified Wright-Giemsa protocol (Sigma-Aldrich), and representative images were acquired on an Olympus BX46 microscope with an Olympus DP72 camera at ×50 magnification with a 0.9 aperture. Olympus cellSens Standard 1.5 image acquisition software was used.

### Flow cytometry

Diagnostic flow cytometric analysis was performed as previously described.[4] Data were analyzed using FlowJo Version 9.3.3 software (TreeStar).

### RT-PCR and Flt3 expression analysis

RNA was extracted from whole bone marrow, thymus, or lymphnode where appropriate using TRIzol (Invitrogen), and reverse transcribed using the iScript cDNA Synthesis Kit (Bio-Rad). Quantitative RT-PCR was performed using the iCycler iQ multicolor real-time PCR system (Bio-Rad), and transcript levels were normalized to *Rps16*. Primers spanning exons 16 and 17 were used to detect *Flt3* expression (Table S1).

### Loss of heterozygosity (LOH) analysis of Flt3 allele

Genomic DNA was extracted from tail tissue, whole bone marrow, thymus, or lymph node where appropriate, using the Wizard Genomic DNA Purification Kit (Promega). PCR amplification was performed using 50mg of genomic DNA with *Flt3* genotyping primers flanking the ITD mutation (Table S1).

### Transplantation

1x10^6^ whole bone marrow cells from leukemic mice were transplanted into sub-lethally irradiated C57Bl/6-CD45.1 recipients (7.5 cGy) by tail vein injection. Blood was collected from the facial vein every 3-4 weeks for CBCs and flow cytometric analysis of engraftment. Additionally, recipients were monitored for visible signs of disease development.

### Stem cell compartment

Eight weeks post pIpC injection, whole bone marrow was isolated, and stem cell and progenitor populations were quantified as previously described.[18] Phenotypic definitions of these compartments are as follows: LSK: Lin^−^Sca-1^hi^c-KIT^hi^; LT-HSC: Lin^−^Sca-1^hi^c-KIT^hi^CD34^−^CD135^−^; ST-HSC: Lin^−^Sca-1^hi^c-KIT^hi^CD34^+^CD135^−^; MPP: Lin^−^Sca-1^hi^c-KIT^hi^CD34^+^CD135^+^; CMP: Lin^−^Sca-1^−^c-KIT^hi^CD34^+^CD16/32^mid^; GMP: Lin^−^Sca-1^−^c-KIT^hi^CD34^+^CD16/32^hi^; MEP: Lin^−^Sca-1^−^c-KIT^hi^CD34^−^CD16/32^−^.

### Colony formation assays

Bone marrow was isolated from mice 8 weeks post pIpC injection, and subjected to lineage depletion using the MACS cell separation system (Miltenyi Biotec). 10,000 lineage negative bone marrow cells were plated on Methocult M3434 (Stemcell Technologies), and analyzed at day 9-11. Colonies were disaggregated, and 200 cells were re-plated and scored for subsequent secondary and tertiary colony formation. Three mice were assayed per genotype, and experiments were performed in triplicate.

## SUPPLEMENTARY MATERIAL


