# Supplementary material for: Dnmt3a deletion cooperates with the Flt3/ITD mutation to drive leukemogenesis in a murine model
Source: Oncotarget. 2016 Sep 12;7(43):69124–35. doi: 10.18632/oncotarget.11986 (PMC5342464; doi:10.18632/oncotarget.11986)
Supplement: Supplementary file 1 [file oncotarget-07-69124-s001.pdf]

Dnmt3a deletion cooperates with the *Flt3/ITD* mutation to drive leukemogenesis in a murine model

Supplementary Material

Table S1. Primer sequences used for genotyping, verification of LoxP recombination, and RT-PCR

	**Primer**	**Sequence**
**Genotyping**	ITD-F	TGC AGA TGA TCC AGG TGA CT
	ITD-R	CTC TCG GGA ACT CCC ACT TA
	Flox-F	AGG AAG TCG ATG TTG GCA CT
	Flox-R	TGA TGC ACT GCT TGT CTT CC
	Dnmt3a-F	TGG GGA TTT GAG AGG TGA AG
	Dnmt3a-R	GTG GAG CAC TGA ACA GCA AG
	Mx1-Cre-F	ATG TTC AAT TTA CTG ACC G
	Mx1-Cre-R	CGC CGC ATA ACC AGT GAA AC
	Dnmt3a LoxP-F	TGG GGA TTT GAG AGG TGA AG
	2LoxP-R	AAG CCT CAG GCC CTC TAG GCA AGA T
	1LoxP-R	TGA GTG GTG AGG CCC AGC TTA TCG A
**qPCR**	Flt3-F	TGG GAC ACC ATG ACA ACA TC
	Flt3-R	GGA ATT TGA ATG TGC CTG GA
	mS16-F	CCA ATT TGA ATG TGC CTG GA
	mS16-R	ACT GAG GTG TGG CTG TGA TG

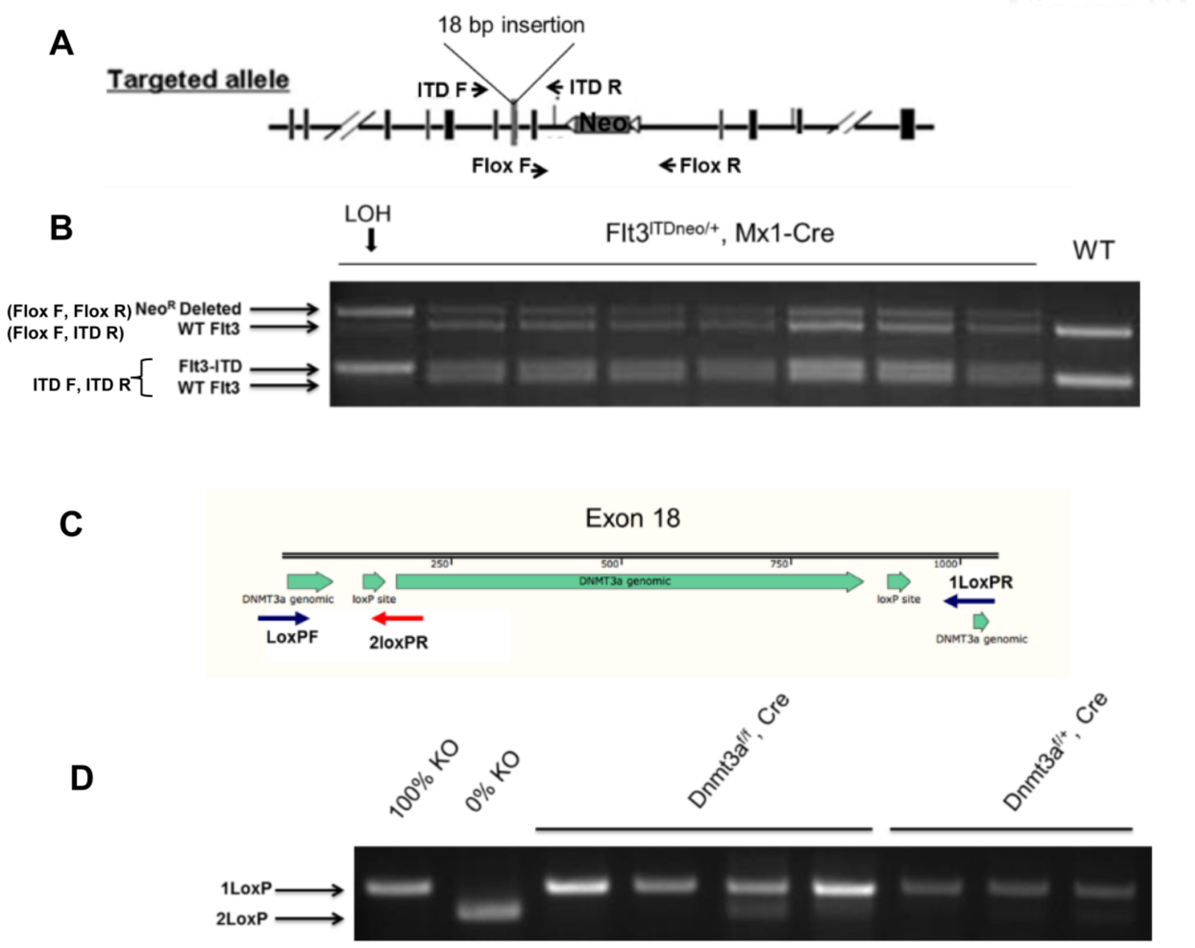

Figure S1. Confirmation of LoxP recombination after pIpC injection.

(**A**) Schematic of the targeted *Flt3-ITD* allele. An 18 bp ITD was inserted into exon 14, with a LoxP-flanked PGK-Neo cassette inserted downstream. Two sets of primers were used to amplify genomic DNA extracted from bone marrow 8 weeks post pIpC injection. (**B**) Gel depicting a wild type mouse (right), heterozygous *Flt3*$^{ITD/+}$ mice, that are positive for excision of the PGK-Neo cassette (center), and an example of a mouse exhibiting LOH, where amplification of the wild type allele is absent. (**C**) Depiction of exon 18 of *Dnmt3a* flanked by LoxP sites, and relevant primers used to detect Cre mediated recombination. 1LoxPR is designed to specifically

amplify the targeted allele, as it is complimentary to DNA remaining from the targeting vector (**D**) Gel electrophoresis of products to determine Cre mediated excision of exon 18 within the targeted Dnmt3a alleles. 100% knocked out (100% KO) control DNA was kindly provided by Dr. Christopher Gamper. 0% knocked out (0% KO) control DNA was extracted from Dnmt3af/f; Mx1-Cre- murine bone marrow.
